# Species perceived to be dangerous are more likely to have distinctive local names

**DOI:** 10.1186/s13002-021-00493-6

**Published:** 2021-12-11

**Authors:** Harith Farooq, Cláudio Bero, Yolanda Guilengue, Clementina Elias, Yasalde Massingue, Ivo Mucopote, Cristóvão Nanvonamuquitxo, Johan Marais, Alexandre Antonelli, Søren Faurby

**Affiliations:** 1 Gothenburg Global Biodiversity Centre, Göteborg, Sweden; 2 Department of Biological and Environmental Sciences, University of Gothenburg, Göteborg, Sweden; 3 Faculty of Natural Sciences, Lúrio University, Cabo Delgado, Mozambique; 4 African Snakebite Institute, Pretoria, South Africa; 5 Royal Botanic Gardens, Kew, Surrey UK; 6 Department of Plant Sciences, University of Oxford, Oxford, UK

**Keywords:** Mozambique, Amphibians, Reptiles, Snakes, Snakebite, Indigenous, Local knowledge

## Abstract

**Background:**

Species with direct uses, such as sources of food, shelter, building material and medicine tend to have more specific local names. But could the same apply for species that people fear?

**Methods:**

To address this question, here we explore the behavior and perception of species diversity and dangerousness through a survey of 1037 households in nine villages in Cabo Delgado, northern Mozambique. We compare people’s knowledge of snakes with that of lizards and amphibians.

**Results:**

We find that northern Mozambicans know four to five times more local names for snakes than for lizards and frogs, despite the local species richness of snakes being comparable to the diversity of lizards and frogs. We further find that local knowledge was on par with the academic literature regarding snakebite symptoms.

**Conclusions:**

Our results suggest that fear can increase the level of specificity in naming species among indigenous communities, which could lead to biases in the mapping and protection of species that include data from citizen reports.

**Supplementary Information:**

The online version contains supplementary material available at 10.1186/s13002-021-00493-6.

## Background

Scientific knowledge of biodiversity is affected by several biases, such as those associated with accessibility, organism group, and overall socio-economic-political conditions that enable access to fieldwork and information [1–4]. Similarly, local knowledge of biodiversity may tilt toward species that exhibit peculiar characters such as appearance, habitat, or utility [5, 6], including factors such as medical importance and economic value [7].

Here we refer to Indigenous and Local Knowledge (ILK) as the accumulation, practices and beliefs about the natural world that are transmitted through generations, [8]. One well-studied aspect of ILK is the salience of species—the set of known species in a community, which also tends to be biased toward peculiarities [9]. Unlike the scientific naming process, ILK may consider a diversity of species that is uncorrelated with the number of local names. Local communities may, for instance, recognize that there are more than one species under a local name [10], although the concept of species is not universal.

Humans interact with and change their environment in a variety of ways depending on their culture, needs and practice. Therefore, understanding the relationship between humans and the co-existing biodiversity is crucial to the implementation of effective conservation strategies. Ethnozoologists, for instance, have provided fundamental contributions to our understanding of conservation and long-term human survival, given the traditional dependency of many societies on co-occurring animals for resources [11] as well as cultural (e.g., myths and legends) importance [12].

One additional but less studied aspect that may influence our understanding of the diversity and distribution of the world’s species is their threat to people. Knowledge of which species are potentially dangerous or even lethal should have a direct effect on the reproductive success of people and therefore be strongly selected for. A primary example of a threat that could influence biodiversity knowledge is that posed by snakes. Among over 3800 species of snakes known worldwide [13], over 200 are considered potentially dangerous to humans by causing death and/or permanent damage [14, 15].

Snakebites are of particular public health importance in Africa and other tropical areas, and therefore considered a category A neglected tropical disease. This category established by the World Health Organization refers to infectious substances capable of causing permanent disability, life-threatening or fatal disease to humans or animals [16]. It is estimated that up to 5.5 million snakebites occur annually around the world, resulting in around 1.8 million cases of snakebite envenoming and 94,000 deaths [17, 18].

Snakebite mortality affects mostly poor people, associated with poorly constructed housing, and with limited access to education and health care [19, 20]. Snakebites push poor people further into poverty due to high treatment costs, enforced borrowing and loss of income [20]. They represent an occupational and environmental injury that mainly affects the youth and agricultural workers. Data from South Asia suggest that the mean age of snakebite victims is around 30 years, and three-quarters of the victims are in the 10- to 40-year aged group (broadly agreeing with the demography), who comprise the most productive members of rural communities [21] and could also affect human reproductive success in some areas.

Snakebites are a large but likely substantially under-reported problem in sub-Saharan Africa [18]. They cause an estimated 20,000 to 32,000 annual deaths in the region [20]. Although high, with figures similar to yellow fever (29,000–60,000 deaths [22]), these numbers may still reflect under-reporting from many parts of this region.

Mozambique is home to at least 14 snakes of medical importance (Table 1), i.e., snakes that can potentially cause death or limb amputation [15]. The presence of potentially dangerous snakes may result in their killing, as observed in most continents [23–29] and ultimately impact their conservation. In addition to housing more than a dozen dangerous snakes, about 70 percent of Mozambique’s population live in rural areas and obtain their livelihood from agriculture [30], which in turn exposes millions of people to snakebites. However, to our knowledge, no data have been published concerning snakebites in the country [15, 18]. In this study, we surveyed 1037 households in nine communities of the province of Cabo Delgado, Northern Mozambique. Based on confirmed species occurrences and global species distribution modeling of all medically important snakes [15], we consider that seven species occur in our study area: Bibron’s Stilleto Snake*,* Puff Adder, Black Mamba, Boomslang, Mozambique Spitting Cobra, Forest Cobra and Mozambican Vine Snake (Table 1).

**Table 1:** Snakes of medical importance in Mozambique

Family	Common name	Scientific name	Expected to occur in the study area
Atractaspidae	Bibron’s Stilleto Snake	*Atractaspis bibronii* Smith, 1849	*X*
Viperidae	Puff Adder	*Bitis arietans* (Merrem, 1820)	*X*
Viperidae	Gaboon Viper	*Bitis Gabonica* (Duméril, Bibron & Duméril, 1854)	
Viperidae	Swamp Viper	*Proatheris superciliaris* (Peters, 1855)	
Elapidae	Green Mamba	*Dendroaspis angusticeps* (Smith, 1849)	
Elapidae	Black Mamba	*Dendroaspis polylepis* Günther*,* 1864	*X*
Elapidae	Rinkhals	*Hemachatus haemachatus* (Bonnaterre, 1790)^a^	
Elapidae	Snouted Cobra	*Naja annulifera* Peters, 1854	
Elapidae	Forest Cobra	*Naja subfulva* Laurent, 1955^b^	*X*
Elapidae	Black Necked Spitting Cobra	*Naja nigricollis* Reinhardt, 1843	
Elapidae	Mozambique Spitting Cobra	*Naja mossambica* Peters, 1854	*X*
Colubridae	Boomslang	*Dispholidus typus* (Smith, 1828)	*X*
Colubridae	Southern Vine Snake	*Thelotornis capensis* Smith, 1849	
Colubridae	Mozambican Vine Snake	*Thelotornis mossambicanus* Smith, 1849	*X*

^a^There are no confirmed collections of Rinkhals (*Hemachatus haemachatus*) in Mozambique, but it has been collected in Zimbabwe near the border with Mozambique, so the species likely occurs within Mozambique as well

^b^Corrected from *N. melanoleuca* [15] to *N. subfulva* following Wüster, Chirio [31]

**Fig. 1:**
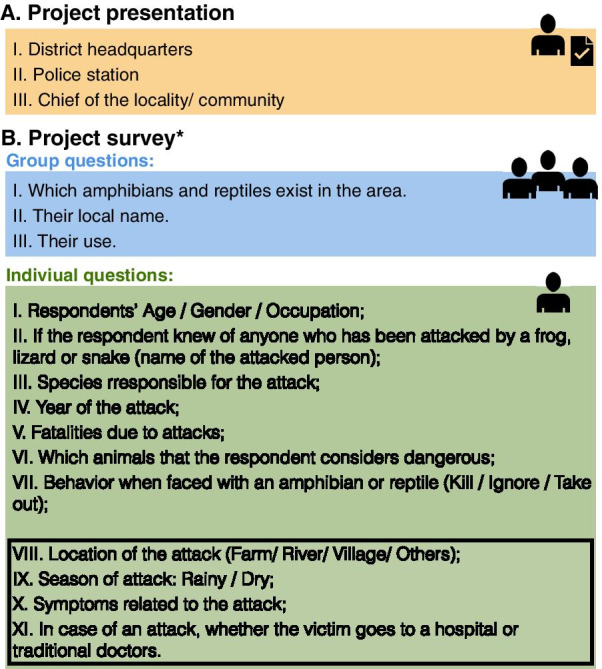
Infographic of the structure of the survey used in this study. After we presented and received authorization from the district headquarters, police station and chief of site we conducted the interviews. Since the awareness of amphibian and reptiles is held by few individuals in the study sites, we decided to interview the site as a whole only in regard to the information on common names and their use

We investigate whether communities in rural areas without any access to biodiversity literature can identify and distinguish between species within the two major herpetofauna groups: amphibians and reptiles. We additionally describe communities’ behavior toward amphibians and reptiles when encounters occur in houses, villages or the woods. This study is based on novel surveys of 1037 households in nine communities of the province of Cabo Delgado, Northern Mozambique (Fig. 1).

## Methods

### Study site

Cabo Delgado is Mozambique’s northernmost province. The coast consists of the ecoregion Southern Zanzibar-Inhambane coastal forest mosaic, while on land the prevalent ecoregion is the Eastern miombo woodlands [32]. Cabo Delgado consists of a mix of lowland areas and inselbergs rising up to 1200 m asl. The yearly average is 30 degrees Celsius (86° F), and most precipitation takes place between December and April, when monthly rainfall averages between 125 and 225 mm per day [33].

### Survey methodology

**Fig. 2:**
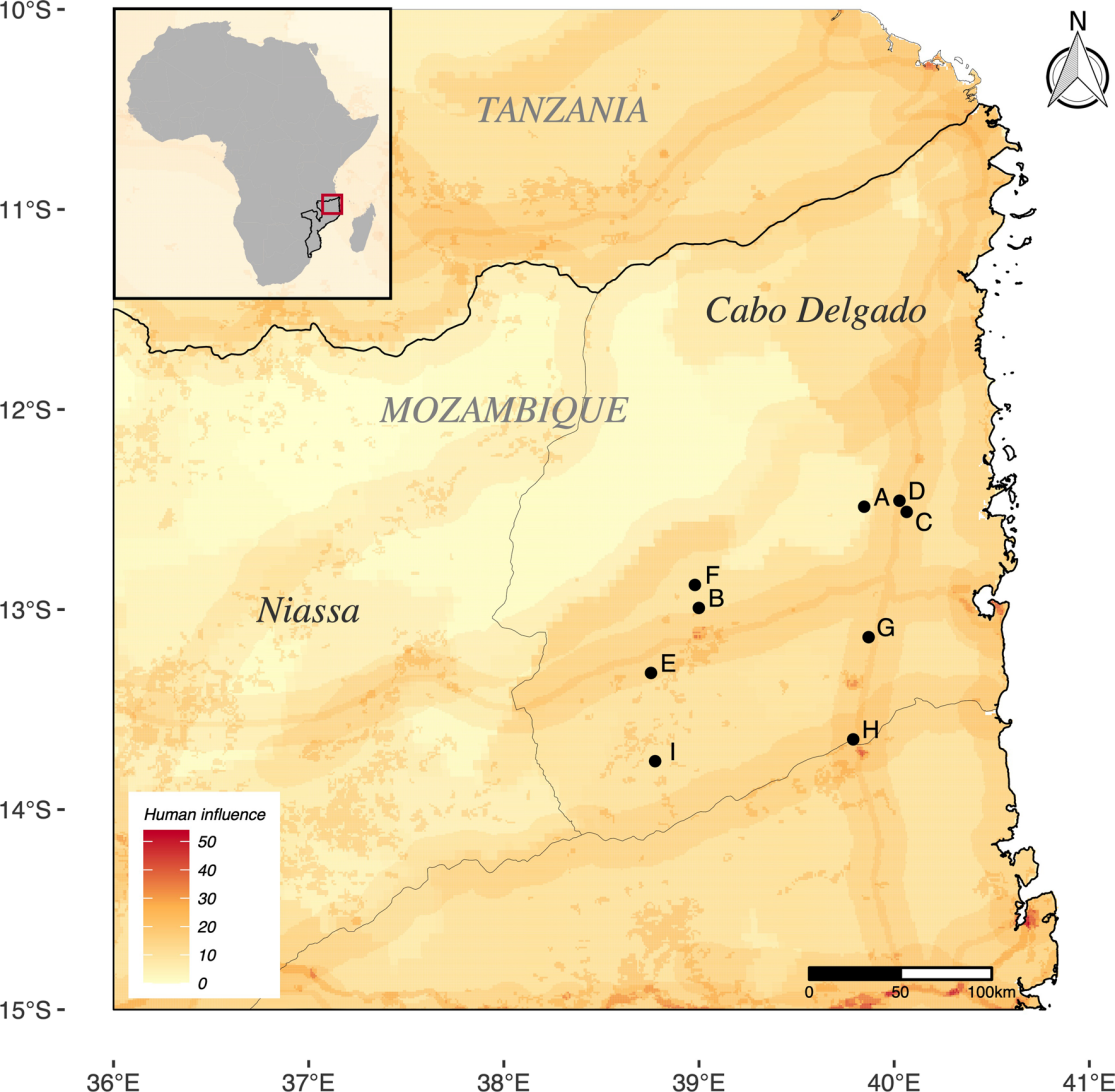
Communities surveyed in this study: (A) Citate, (B) Eduardo Mondlane, (C) Mitambo, (D) Muaguide, (E) Muapé, (F) Niuhula, (G) Ntique, (H) Ocua, (I) Shopa. In total, we surveyed 1937 households in nine communities situated mostly in the south of the province of Cabo Delgado. The Global Human Influence Index is created from nine global data layers covering human population pressure, human land use and infrastructure, and accessibility. It ranges from 0 (lowest) to 64. The range of human influence in our sites ranged from 4 (Citate) to 18 (Muaguide and Ocua)

The survey (Fig. 1) was conducted in nine communities in Cabo Delgado, northern Mozambique (Fig. 2, Additional file 1: Table S1), between the 15th of July and 13th of August of 2019. Members of our team collected information on perceptions and behavior toward amphibians and reptiles as well as attacks in the last 20 years. In total, 1037 out of 1539 households in the villages were surveyed (67%). We interviewed people between the ages 15 and 87 (median = 35) and 60% were female. Of all the interviewees, 97% practiced agriculture exclusively and 2% practiced agriculture and some other activity. Only 1% carried out activities unrelated to agriculture such as teaching or carpentry (Additional file 1: Table S1).

For the survey and analyses, we exclude information on crocodilians and turtles and divide the remaining reptiles into “snakes” and “lizards”—the latter being the term we use for all species in the order Squamata excluding snakes. We exclude crocodiles and turtles from the survey since both groups only contain one species in the region and therefore cannot be used to understand whether or not similar organisms share local names. The reasoning behind this grouping is that snakes are the only squamates in the region that are medically important. We acknowledge that the use of non-monophyletic terms are undesirable in evolutionary studies, but animals grouped here as “lizards” are morphologically and genetically more diverse than snakes.

We identified medically important snakes based on the classification by Longbottom, Shearer [15]. Our survey shows that all but the Vine Snake were mentioned by the communities as responsible for attacks. When discussing the surveys, we will use the term “reported” for symptoms obtained by the communities, and “literature symptoms” for symptoms discussed in the scientific literature. To conduct this study, we received authorization from the head of the districts of Ancuabe, Balama, Chiure, Meluco, Montepuez and Namuno as well as local support from the Chiefs of the villages of Citate, Eduardo Mondlane, Mitambo, Muaguide, Muapé, Niuhula, Ntique, Ocua and Shopa. The stamped permits are available upon request.

To assess the list of species occurring in the study area, we showed people a list of species and photographs possible to occur in the area. We then recorded the presence/absence record as well as the species names in the local language of the region (Makhuwa), hereafter referred to as local names and their local use. Two of the co-authors in this study are native speakers of Makhuwa, and therefore, there was no need for translators. The list of species with their local names, use and the source of photographs used in the survey, are available in Additional file 2: Table S2.

To assess information on attacks, we retrieved information on bite incidents that occurred the in the village which included the species responsible for the attack, the name of the attacked person, the year, the season, location of the attack, the symptoms and finally whether the victim was treated in a hospital, by traditional medicine and if the incident was fatal. To avoid overestimating attacks, we excluded duplicated reported attacks and attacks in which the interviewee was unable to recall the name of the attacked person. To assess the behavior toward the encounters with snakes, lizards and amphibians we asked whether the individuals living in the household would kill, take out, or ignore the animals when they faced them at home, at the village or in the field. The questions VIII–XI fall into a different scope and therefore will not discussed in this study (Fig. 1).

## Results

**Fig. 3:**
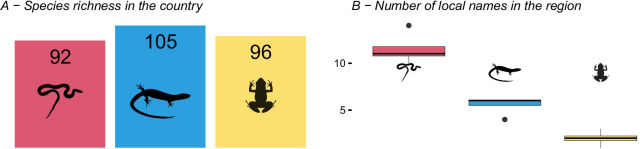
**A** Country-richness of snakes, non-snake reptiles and amphibians [34]. **B** Number of species names by snakes, non-snake reptiles and amphibians. Even though the numbers of species of snakes, non-snake reptiles and amphibians are similar, there are 4 to 5 more names for snake’s species than for other reptiles and amphibians

Our results show that even though the country-level species richness of snakes, lizards and amphibians is similar (92 snakes, 128 lizards and 96 amphibians) (Fig. 3A), communities have on average considerably more names for species of snakes [11] when compared to other reptiles [3] and amphibians [2] (Fig. 3B).

To uniformize the spelling of local names, we used the spelling in [35] whenever possible, but in some instances there were no equivalent matches or some names appeared to be generic names for many species (Table 2).

**Table 2:** Table of local names and use

Scientific name	English common name	Local name
Snakes		
*Atractaspis bibronii* Smith, 1849	Bibron’s Stilleto Snake	Etete^a^
*Bitis arietans* (Merrem, 1820)	Puff Adder	Impoma^a^
*Boaedon capensis* Duméril, Bibron & Duméril, 1854	Brown House Snake	Hiriri^b^/Etatamahuku^a^/Nhanhapa^a^
*Causus defilippii* (Jan, 1863)	Snouted Night Adder	Ivili
*Dendroaspis polylepis* Günther*,* 1864	Black Mamba	N`Rapa^a^
*Dispholidus typus* (Smith, 1828)	Boomslang	Muikomea^a^
*Elapsoidea boulengeri* Boettger, 1895	Boulenger’s Garter Snake	Ihakani^a^
*Hemirhagerrhis nototaenia* (Günther, 1864)	Eastern Bark Snake	Namunhapa^c^
*Naja subfulva* Laurent, 1955^b^	Forest Cobra	N’tawe
*Naja mossambica* Peters, 1854	Mozambique Spitting Cobra	M’rhawe
*Natriciteres Olivacea* (Peters, 1854)	Olive Marsh Snake	Kaputi^a^
*Philothamnus* sp. Smith, 1847	–	Namanthapa
*Psammophis mossambicus* Peters, 1882	Olive Whip Snake	Nalu
*Psammophis orientalis* Broadley, 1977	Eastern Stripe-bellied Sand Snake	Ilumathanu^d^
*Python natalensis* Smith, 1840*	African Rock Python	Ekhuka
*Telescopus semiannulatus* Smith, 1849	Tiger Snake	Nantxuwa^a^/Ntupessa^a^
*Thelotornis mossambicanus* (Bocage, 1895)	Mozambique Twig Snake	Niwiwirhi
Typhlopidae indet	Worm snakes	Txua^a^/Ethokathoka
Lizards		
*Agamidae/Cordylidae*	–	Nikuthukuthu
*Chamaeleo dilepis* Leach, 1819	Flap-necked Chameleon	Namanria
*Lygodactylus grotei* Sternfeld 1911	Grote’s dwarf gecko	Nakoko^e^
*Matobosaurus validus* (Smith, 1849)	Common Giant Plated Lizard	Namakwakwa
*Mochlus sundevallii* (Smith, 1849)	Sundevall's Writhing Skink	Nantukutuvili^a^
*Trachylepis* sp. Fitzinger, 1843	–	Hekwatxo^a^
*Varanus* sp.** Merrem, 1820	Monitor lizard	Ihala
Amphibians		
*Breviceps mossambicus* Peters, 1854	Mozambique rain frog	Ihenene
*Pyxicephalus edulis* Peters, 1854**	Edible Bull Frog	Nume^a^
*Xenopus muelleri* (Peters, 1844)	Müller's Clawed Frog	Naphulu
General term for all other frogs	–	Marapi
Other***		
*Kinixys* sp. Bell, 1827	Hinge-back Tortoise	Khapa
*Crocodylus niloticus* Laurenti 1768	Nile Crocodile	Ekonya

^a^The name did not match the listed name in the literature

^b^Same name as the Bibron’s Stilleto Snake in the literature

^c^The name is referred to Boulenger’s Garter Snake

^d^Name missing in the literature

^e^General term for lizard

*Skin is used to make shoewear and drum

**Edible species

***These species were not included when in any of the analysis in this study

**Fig. 4:**
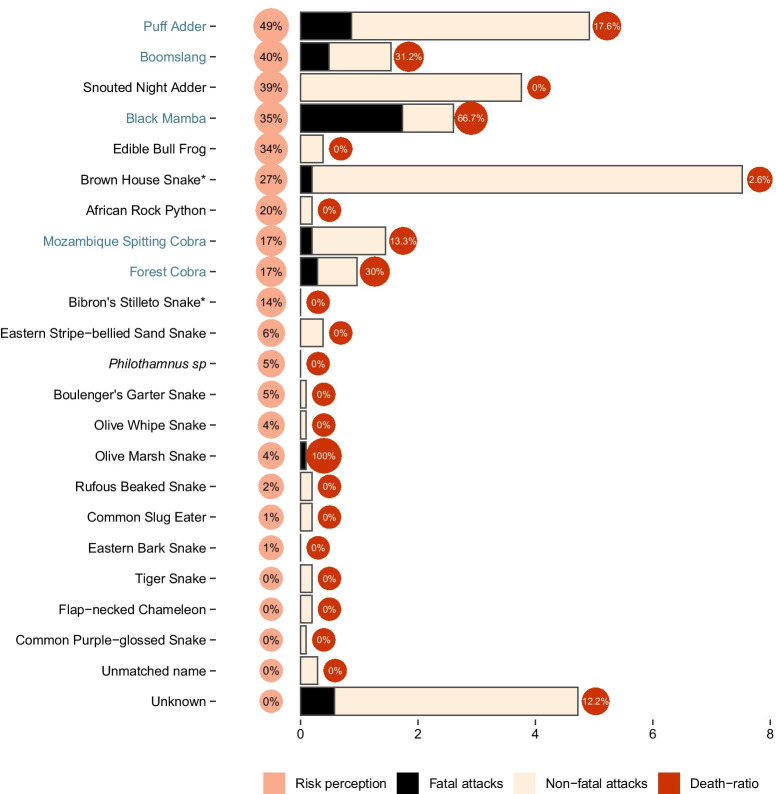
Comparison between species perceived to be dangerous (Risk perception) and attacks per surveyed household (*N* = 1037) and death-ration of the attacks. Species in blue highlight the medically important snakes. The fatal attacks attributed to the Olive Marsh Snake (*Natriceres olivacea*) are most likely a result of a misidentification since the species is known to be harmless. *One of the local names attributed to the Brown House Snake (*Boaedon capensis*)—Hiriri, coincided with the Bibron’s Stilleto Snake (*Atractaspis bibronii*) in the literature. Therefore, we expect a great proportion of the attacks of the Brown House Snake to have been caused by the Bibron’s Stilleto Snake, mostly because of the reported symptoms which included pain, swelling and hounds and these are known to be caused by the Brown House Snake. The species with most attacks on humans was the Brown House Snake (*Boaedon capensis*), the deadliest snake was the Black Mamba (*Dendroaspis polylepis*) and the African Edible Bull Frog (*Pyxicephalus edulis*) ranked 5th on the species perceived to be dangerous even though virtually no attacks were attributed to amphibians

We found that the snake species blamed for most attacks was the Brown House Snake, followed by the Black Mamba and Puff Adder (Fig. 4). In terms of fatal attacks, the snake most claimed for deaths was the Black Mamba, where we reported casualties in more than 1.7% of the households in the last 20 years, followed by the Puff Adder with 0.87%, then the boomslang and the cobra (Fig. 4). The highest ratio of deaths by attack was attributed to the Black Mamba with 67% of the bites (Fig. 4). The snake most mentioned as dangerous was the Puff Adder, followed by the Boomslang, Snouted Night Adder (*Causus defillipi*) and the Black Mamba (Fig. 4). The African bull frog (*Pyxicephalus edulis*) and the brown house snake were mentioned more times as dangerous than the Mozambique Spitting Cobra, African Rock Python and the Bibron’s Stiletto Snake (Fig. 4).

**Fig. 5:**
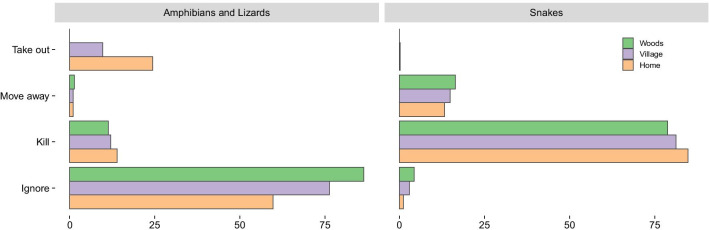
Percentage per household of people’s behavior toward non-snake reptiles and amphibians versus snakes in houses in the villages and the woods. The x-axis is the percentage of households that reported each behavior. Over 75% of the households actively kill snakes, even when the encounters happen in the woods, while non-snake reptiles and amphibians are usually ignored, even inside their houses

When the community surveyed is faced with amphibians and reptiles, snakes are often killed, regardless where the encounter took place (Fig. 5).

Knowledge of symptoms caused by the different species of snakes occurring in the area was on par with the existing literature. A full table of species and their reported and literature-based symptoms is provided in the Additional file 2 (Additional file 2: Table S3).

## Discussion

Our study shows there were four to five times more local names for snakes than other reptiles or amphibians. Even snakes that were deemed harmless by the local communities had local names. It is plausible to interpret that the overall fear of snakes is the key driver behind this naming pattern. It has been shown that the existence of local names for different species does not directly translate to how many species are in reality recognized by indigenous people [10] and nor how well people understand their ecology [36].

We argue that names for each species are highly beneficial when communicating about them, for example, when informing a local healer which species is responsible for a snakebite, or when teaching children about which animals are potentially dangerous. While lexical diversity may not adequately capture the magnitude of local knowledge about different organism groups, we argue that it may still be informative about the society’s need to communicate details about the different groups.

Many species may be extremely difficult to tell apart and may therefore lack local names. But in our study, we found no indication that local snakes are easier to tell apart than it is for amphibians or lizards. We would expect species belonging to the same genus or perhaps family to be harder to tell apart. But local amphibians are represented by 13 families (12 families of anurans, i.e., frogs and toads and one family of limbless borrowing caecilians), lizards are represented by 10 families and snakes by 13 families. Since the number of families is very similar, we would expect in the case of no differentiation between dangerous or non-dangerous animals, that we would find similar number of species names.

### Taxonomical considerations

Taking into account the fact that even trained biologists can misidentify species [37], we expect a degree of uncertainty from the identifications from locals. In many situations, it may be hard to get a full glimpse of the snake when it is dark or when they happen to be half-hidden. While most of the symptoms described by bites from the various species match the reports in the literature, and the species were therefore with high likelihood correctly identified by the locals, there are a few exceptions discussed below.

Based on the descriptions for some of the bites provided by local people, and taking into consideration morphological similarities among certain species, we expect that bites reported from the Common Slug Eater (*Duberria lutrix)* and the Rufous Beaked Snake (*Ramphiophis rostratus)* are caused instead by the Olive Whip Snake (*Psammophis mossambicus)*. Similarly, deadly bites from Brown House Snakes or Olive Marsh Snakes might be caused by any of these: Black Mamba, Forest Cobra or Mozambique Spitting Cobra. The thread snakes from the family Typhlopidae are unable to bite, and were most likely mistaken with the Bibron’s Stiletto Snake. Furthermore, the provided local name for the Brown House Snake matched the Bibron’s Stiletto Snake in [35]. This would explain both the high number of Brown House Snake bites and the low number of the Bibron’s Stiletto Snake. The symptoms reported in these bites, usually swelling and hounds, are typical symptoms from Bibron’s Stiletto Snake bites.

Some species can be hard to tell apart, such as in the case of the Forest Cobra and the Mozambique Spitting Cobra, but the communities still provided different local names for each of the species. Nevertheless, the reported cases by any *Naja* sp. may belong to any of the two species. Also, the Snouted Night Adder (*Causus defilippii*), even though not considered medically important in Longbottom, Shearer [15], accounts for a painful bite that causes swelling [38] and was understandably recognized as the third most dangerous animal.

### Local knowledge and behavior

Our results highlight the higher cultural importance of snakes than other herpetofauna. We interpret the high number of species names of snakes (Fig. 2B), compared to other herpetofauna, as a consequence of the existence of medically important snakes in the area. Since the dangers are only associated with some snake species, it may be vital for communities to communicate which dangerous snake bit someone, but also to correctly identify harmless species to avoid spending unnecessary energy in trying to kill or remove them. Supporting this interpretation, we note that the East African bull frog (*Pyxicephalus edulis*) was the only amphibian perceived as dangerous, although the species is also edible which may also contribute to it having a local name. Our study revealed two accounts of attacks caused by this species, which has been suggested to cause a painful bite [39]. It is therefore highly interesting that this is one of only the three amphibians (among the 97 species in the country) that had a local name (the other two are the highly aberrant rain frogs of the genus *Breviceps*, and the clawed frogs of the genus *Xenopus*).

Overall, our results on high local knowledge among rural communities’ contrast with previous reports. Studies conducted in patients at a hospital in KwaZulu-Natal in South Africa suggested that 60% of patients were unable to identify the snake using photographs, and 7% of identifications were inaccurate [40]. To explain this difference, we speculate that the questionaries conducted at hospitals may have been biased toward people living in more urbanized environments [40], while isolated rural communities with limited access to health centers may require—and indeed possess—a considerably better understanding of snakes and how to treat their bites.

In terms of behavior, when community members are faced with amphibians and reptiles, snakes are often killed, even when the encounters take place in the woods. This may be a consequence of preventive behavior. The killing of snakes has been reported in all continents except Antarctica [23–29] and is associated with fear, disgust and myths [29]. Therefore, initiatives focused on snake diversity and distinguishing between dangerous and non-dangerous snakes should be co-produced with rural communities to reduce negative emotions and increase tolerance. This would ultimately contribute to prevent the indiscriminate intentional killing of snakes and promote their conservation [29]. Such initiatives may further also reduce the number of attacks, since aggressive behavior toward snakes may be one of the causes of snakebites in the first place [25].

### Perception of danger and bites

Our results highlight a large knowledge about snakes and snakebites by local communities in northern Mozambique. For example, Puff Adder bite symptoms were described by the communities as *“Swelling of the limb where the bite occurred, blisters across the whole limb, fever, excessive pain, difficulty in locomotion, heartrate increase, mostly at night and the pain may last 1–3 months. Fatalities occurred just after the attack.*” The full list of symptoms per species and whether they match the literature can be found in Additional file 2: Table S2.

Eight species, including a chameleon, that have been claimed to cause attacks, have not been considered either medically important by the literature or dangerous by less than 10% of the households in the communities. These numbers are likely related to both misidentification and varying degrees of aggressiveness between snake species, where having medically relevant venom is not correlated with aggression. The snake species most mentioned as dangerous was the Puff Adder, followed by the Boomslang, which at least in South Africa are not considered aggressive. Bites from Boomslangs are very rare according to the available literature [41], amounting to perhaps one or two confirmed bites per year in South Africa [38, 42]. In African savannas, approximately 90% of all bites are attributed to vipers, where the Puff Adder is the most dangerous species and responsible for most bites [43].

In our study, the overestimated non-dangerous Brown House Snakes discussed above, and the snouted night adder were responsible for most attacks and as expected, Black Mambas caused fewer but deadlier attacks when compared to vipers. In terms of deadly attacks, black mambas caused most fatalities by a large extent, followed by Puff Adders and Boomslangs. Since it may take a few days for adults to die from snakebites [38, 44], many of the causalities we report in Northern Mozambique may be fully avoidable, if there were enough antivenom and doctors trained in treating snakebites. Understanding how species, such as the Boomslang that rarely bites, accounted for so many deaths, could potentially help diminishing bites in the region.

In South Africa, the Mozambique Spitting Cobra accounts for the most serious bites, followed by the Puff Adder and Bibron’s Stiletto Snake. Black Mamba bites are quite rare and Brown House Snake bites are rarely documented [45]. There are also a fair number of people bitten by Mozambique Spitting Cobras while asleep in their beds [46]. Puff Adder deaths are rare [47]. They have a slow-acting cytotoxic venom, somewhat similar to Mozambique Spitting Cobra bites, causing progressive swelling, pain, blistering and tissue damage [45]. As reported in this study, the surveyed communities actively kill snakes wherever they encounter them. We believe that snakebite caused by snake’s self-defense to be responsible for a great number of the attacks in the region.

## Conclusions and recommendations

Just as medical importance and economic value, indigenous knowledge is also driven by one of our most instinctive and evolutionary powerful feeling: fear. This may come as no surprise, given that snakebites are a serious issue that can cause death and permanent damage, affecting negatively not only the lives of those attacked by also of their families and communities. Even though there is considerable indigenous knowledge in Northern Mozambique on snakebite symptoms and which species are dangerous, we found that high levels of deaths still result from bites by Black Mambas, Cobras, Boomslangs and Puff Adders (between 67%–18%, respectively). The benefits of attending a health centers in Mozambique following a snakebite have yet to be reported. But data from neighboring countries show less than 1% of deaths resulting from snakebites after patients reach the hospital [40, 48–50], suggesting that snakebite victims may benefit from appropriate medical care.

Based on our findings, we provide three recommendations:

1. Local health centers across the country should have trained staff to deal with snake bites and available antivenom;
2. The creation of accessible literature on snakes and snakebites in the local languages spoken in the country, preferably co-created with rural communities, and including indigenous and local names. This will help reduce unnecessary medical treatments and negative impacts on wildlife populations;
3. An analysis of the snakebite cases that reach the hospitals should be conducted and reported. Important information such as death rates will contribute to clarify how important snakebites are at a national level, and also help improve the current framework of treatment following snakebites at health centers.

## Supplementary Information

**Additional file 1: Table S1.** Raw data from the survey.

**Additional file 2: Table S2.** Sources of photographs. **Table S3.** Symptoms described by the surveyed people in regard to snakebits. **Table S4.** Coordinates and number of surveyed houses in each community.

## Data Availability

All data generated or analyzed during this study are included in this published article [and its supplementary information files].
