# Supplementary material for: Species perceived to be dangerous are more likely to have distinctive local names
Source: J Ethnobiol Ethnomed. 2021 Dec 11;17:69. doi: 10.1186/s13002-021-00493-6 (PMC8665639; doi:10.1186/s13002-021-00493-6)
Supplement: Supplementary file 2 — Additional file 2: Table S2. Sources of photographs. Table S3. Symptoms described by the surveyed people in regard to snakebits. Table S4. Coordinates and number of surveyed houses in each community. [file 13002_2021_493_MOESM2_ESM.docx]

Additional file 2

Additional file 2: Table S2. Sources of photographs

| Group | Source of photographs |
| --- | --- |
| Amphibians | Du Preez and Carruthers (2009) |
| Reptiles | Graham and Marais (2007) |

Additional file 2: Table S3. Symptoms described by the surveyed people in regard to snakebites.

| Snake species | Snakebite symptoms described in the survey | Comments according to published literature |
| --- | --- | --- |
| REPTILIA |  |  |
| COLUBRIDAE |  |  |
| Olive grass snake *(Psammophis mossambicus)* | Swelling of the affected limb that may take up to a month to go back to normal. | Symptoms match the literature (African Snakebite Institute, 2020). |
| Eastern Stripe-bellied Sand Snake *(Psammophis orientalis)* | Swelling of the affected limb. | Symptoms match the literature (African Snakebite Institute, 2020). |
| Boomslang *(Dispholidus typus)* | Darkening of the skin, throwing up, swelling and excessive pain. Increased blood circulation. In fatalities, victims had convulsions after the bite and death occurred up to 24h after the bite. | Symptoms match the literature (Du Toit, 1980), although death is expected to occur in up to 72 hours. |
| ELAPIDAE |  |  |
| Black mamba *(Dendroaspis polylepis)* | Excessive pain, swelling of the affected limb, in some fatal cases the death occurred just after the bite and in others after a few days. In non-fatal bites, victims showed blisters in the affected limb, swelling and individuals were cured after a month. | The literature backs up the high incidence of fatalities that occur short after the bite but as a species with neurotoxic venom, blisters are not expected to occur (African Snakebite Institute, 2020; Müller et al., 2012). Since blisters only showed up on victims that survived the bite, there is a chance that these bites were caused by *Naja mossambica*, a cytotoxic species. |
| *Angolan garter snake (Elapsoidea semiannulata)* | Swelling, pain and blisters in the affected limb. | Symptoms match with the literature described as local pain, swelling and lymphangitis (Müller et al., 2012). |
| Forest cobra (*Naja melanoleuca)* | Swelling in the affected limb, blisters that may last up to 3 months. Fatalities occured in some just after the bite, and for others few days after the bite. | Symptoms match the literature including the possibility of causing fatalities (African Snakebite Institute, 2020; Müller et al., 2012). |
| *Mozambique spitting cobra (Naja mossambica)* | Blindness if the venom gets into contact with the eyes, and when the victim is bitten, there is swelling and excessive pain. | Symptoms match the literature including the possibility of causing fatalities (African Snakebite Institute, 2020; Müller et al., 2012). |
| LAMPROPHIIDAE |  |  |
| Brown house snake *(Boedon capenses)* | In some cases, swelling in the area bitten and pain that may last up to three days. | This species is regarded as completely harmless (African Snakebite Institute, 2020) |
| *Rhamphiophis rostratus* | Swelling and pain in the affected limb ^(a)^. | Very mild and has no effects on humans (African Snakebite Institute, 2020). |
| Viperidae |  |  |
| Puff adder *(Bitis arietans)* | Swelling of the limb where the bite occurred, blisters across the whole limb, fever, excessive pain, difficulty in locomotion, heartrate increase, mostly at night and the pain may last 1-3 months. Fatalities occurred just after the attack. | Most symptoms reported are consistent with the literature (Müller et al., 2012). Fever not being reported may occur as a result of infections from the blisters and wounds. Although fatalities are rare they are known to occur (African Snakebite Institute, 2020). |
| Night adder  *Causus defilippii* | Swelling in the bitten limb, which may last up to a week. The pain is of low intensity when compared to other snakebites. | Both swelling and pain as well as the duration of the symptoms are consistent to the literature (African Snakebite Institute, 2020; Müller et al., 2012). |
| PYTHONIDAE |  |  |
| *Southern African python (Python natalensis)* | Wounds and swelling in the affected limb and in some cases the victims had their lower bodies constricted. | Pythons are non-venomous, but bites can cause serious infections. Attacks on humans are rare as well as fatalities (African Snakebite Institute, 2020; Haacke, 1981). |
| AMPHIBIA |  |  |
| PYXICEPHALIDAE |  |  |
| *Pyxicephalus edulis* | After one of the attacks, swelling in the limb, and difficulties in locomotion that stay for up to two weeks. The other bite recorded had no symptoms. | Bites have been recorded but no symptoms are known to occur (Loveridge, 1950). |

^(a)^ We believe these bites might have been mistaken with *Psammophis mossambicus*

Additional file 2: Table S4. Sites surveyed in this study

| Site | Latitude | Longitude | Number of houses | Enquired houses |
| --- | --- | --- | --- | --- |
| Citate | -12.486 | 39.846 | 245 | 150 |
| Eduardo Mondlane | -12.992 | 38.999 | 222 | 141 |
| Mitambo | -12.512 | 40.064 | 200 | 132 |
| Muaguide | -12.456 | 40.027 | 149 | 108 |
| Muapé | -13.317 | 38.754 | 222 | 141 |
| Niuhula | -12.877 | 38.979 | 65 | 56 |
| Ntique | -13.138 | 39.869 | 133 | 99 |
| Ocua | -13.649 | 39.790 | 136 | 101 |
| Shopa | -13.758 | 38.775 | 167 | 117 |

Additional file 2: Table S5. Behaviour towards species in the field/villages/home

|  | **Amphibians and lizards** | | | **Snakes** | | |
| --- | --- | --- | --- | --- | --- | --- |
| **Villages** | **Ignores** | **Run away** | **kill** | **Ignores** | **Run away** | **kill** |
| Citate | 139 | 0 | 13 | 8 | 36 | 108 |
| Eduardo Mondlane | 115 | 0 | 21 | 3 | 19 | 118 |
| Mitambo | 118 | 3 | 11 | 8 | 17 | 108 |
| Muaguide | 71 | 5 | 16 | 3 | 13 | 75 |
| Muapé | 132 | 0 | 12 | 2 | 26 | 115 |
| Niuhula | 44 | 4 | 8 | 2 | 10 | 44 |
| Ntique | 89 | 1 | 9 | 8 | 14 | 77 |
| Ocua | 95 | 1 | 5 | 9 | 21 | 71 |
| Shopa | 93 | 0 | 23 | 2 | 15 | 101 |

**References**

African Snakebite Institute. (2020). *African Snakebite Institute*. Retrieved Oct, 14, 2020 from [www.africansnakebiteinstitute.com](file:///Users/stella/Downloads/Submission_6_resub/Submission_6_resub/Additional%20files/www.africansnakebiteinstitute.com)

[Record #564 is using a reference type undefined in this output style.]

Du Toit, D. (1980). Boomslang (Dispholidus typus) bite. A case report and a review of diagnosis and management. *South African medical journal SAMJ*, *57*(13), 507-510.

[Record #565 is using a reference type undefined in this output style.]

Haacke, W. D. (1981). A possible further incident of a human as prey of the African rock python (Python sebae).

Loveridge, A. (1950). History and habits of the East African bullfrog. *East African Geographical Review*, *1947*(89), 253-255.

Müller, G., Modler, H., Wium, C., Veale, D., & Marks, C. (2012). Snake bite in southern Africa: diagnosis and management. *Continuing Medical Education*, *30*(10).
